# MiR-526b-3p Inhibits the Resistance of Glioma Cells to Adriamycin by Targeting MAPRE1

**DOI:** 10.1155/2022/2402212

**Published:** 2022-02-14

**Authors:** Lei Qiu, Shuyuan Xing, Fuxin Lang, Qing Bao

**Affiliations:** ^1^Department of Neurosurgery, Zibo Central Hospital, Zibo, Shandong Province 255000, China; ^2^Department of Neurosurgery, Zouping Hospital of Traditional Chinese Medicine, Zouping City, Shandong Province 256200, China; ^3^Department of Neurosurgery, Huantai People's Hospital, Zibo, Shandong Province 256400, China; ^4^Department of Neurosurgery, Wujin Hospital Affiliated with Jiangsu University, Changzhou City, Jiangsu Province 213100, China; ^5^Department of Neurosurgery, The Wujin Clinical College of Xuzhou Medical University, Changzhou City, Jiangsu Province 213100, China

## Abstract

**Background:**

Cell resistance is the main reason for the high mortality in glioma. Adriamycin (ADR) is a treatment drug for glioma and often leads to chemoresistance. Previous studies have confirmed that the abnormal expression of microRNA (miRNA) affects the resistance of glioma cells.

**Methods:**

RT-qPCR and western blot were conducted for detecting miR-526b-3p levels and related protein expressions. CCK8 assay, colony formation, flow cytometry, and Transwell were adopted to assess cell viability, proliferation, apoptosis, and metastasis. Moreover, downstream targets of miR-526b-3p were identified through a dual-luciferase reporter and RNA pull-down analysis.

**Results:**

Nevertheless, miR-526b-3p functions on glioma cell resistance to ADR are not well characterized. This study demonstrated that miR-526b-3p levels were decreased within glioma cells and further decreased within ADR-resistant glioma cells. Then, miR-526b-3p overexpression repressed glioma cell proliferation and invasion while inducing cell apoptosis. Overexpression of miR-526b-3p within ADR-resistant glioma cells obtained similar results, which suggested miR-526b-3p suppressed glioma cell resistance to ADR. Mechanistically, miR-526b-3p targeted MAPKE1 and negatively regulated MAPKE1 expressions. Restoration of MAPKE1 levels reversed miR-526b-3p effects on the glioma process and resistance to ADR.

**Conclusion:**

These results suggest that miR-526b-3p acts as a diagnostic marker in glioma development and therapeutic target of the glioma resistance to ADR.

## 1. Introduction

Glioma is an intracranial tumor that accounts for more than half of intracranial tumors and causes a high mortality rate [1]. At present, the treatment of glioma is mainly surgery, radiotherapy, chemotherapy, or combination therapy [2]. With the development of these treatment strategies, glioma treatment has been improved. However, the survival rate is still unsatisfactory [3]. Resistance of the glioma cells to chemotherapy is the primary reason for the poor prognosis [4]. Hence, exploring the target to overcome the resistance of the glioma is necessary. Adriamycin (ADR), also called doxorubicin (Dox), is the antibiotic used in tumor therapy, including glioma [5]. The treatment effect of ADR is weakened because of the resistance in glioma. However, the mechanism of ADR resistance in glioma is still unclear. Therefore, we explore the molecular mechanism of the glioma resistance to ADR and expect to find new targets and pathways to decrease the ADR resistance of glioma.

MicroRNAs (miRNAs) exert vital impacts on various physiological and pathological processes through modulating mRNA degradation or posttranscriptional levels of targeted RNAs [6]. Nowadays, growing research has shown that miRNAs' abnormal expression is involved in the resistance of malignancies [7, 8]. For example, miR-204 overexpression inhibits hepatocellular cancer's drug resistance and metastasis [9]. As a tumor suppressor miRNA, MiR-181b has less expression in human glioma and enhances chemosensitivity of TMZ [10]. The recovery or knockout of multiple miRNAs has also been shown to reduce or improve chemoresistance in glioma. MiR-195 levels are decreased within TMZ-resistant glioma cells, and miR-195 upregulation reverses TMZ resistance through regulating cyclin E1 expression [11]. Compared with parental cells, the expression of miR-125b is diminished in TRAIL-resistant glioma cells and increased miR-125b expression resensitizes glioma cells to TRAIL [12]. MiR-127 downregulation significantly increased glioma cell sensitivity to ADR [13]. Nevertheless, the potential role of miRNA in glioma ADR resistance was still largely unknown. Therefore, finding key miRNAs for glioma ADR resistance was of great value to glioma treatment and prognosis.

MiR-526b-3p is considered a tumor suppressor gene involved in multiple biological processes of malignant tumors, including colorectal cancer and cervical cancer [14–16]. In glioma, miR-526b-3p has been reported as a prognosis factor and regulates the tumor process through WEE1 [17]. However, the functions of miR-526b-3p in resistance have not been elucidated.

This study investigated the roles and molecular mechanisms of miR-526b-3p within glioma resistance to ADR. Our findings showed that miR-526b-3p levels were decreased within glioma and ADR-resistant glioma samples. Furthermore, miR-526b-3p upregulation suppressed cell proliferation and cell metastasis, enhancing cell apoptosis. Meanwhile, miR-526b-3p overexpression reduced glioma cell resistance to ADR. Further study confirmed that miR-526b-3p increased the glioma cells' sensitivity to ADR by regulating the expression of MAPRE.

## 2. Materials and Methods

### 2.1. Tissues Sample

All samples in this study were obtained from the Wujin People's Hospital. Glioma tissues and the adjacent normal tissues came from patients that clinically confirmed glioma and accepted the surgical treatment. The patients were treated with ADR and divided into the ADR resistance and nonresistance groups. The tissues were removed and frozen in −80°C refrigerator immediately. All patients who participated in the study were informed to sign the consent. The study was approved by the ethics committee of Wujin People's Hospital and conducted under its supervision.

### 2.2. Cell Culture

Glioma cells T98G, U87, SHG44, and U251 and nonneoplastic glioma cell line H4 were purchased from ATCC (USA). T98G cells were cultured within MEM medium (Minimum Eagle's medium). U87 and U251 cells were cultured within MEM-EBSS medium. SHG44 and H4 cells were, respectively, cultured in the RPMI-1640 and DMEM-H medium. All media were purchased from the Thermo-Scientific. These media were added to 10% FBS (Gibico, NY, USA), and all cells were incubated within the humid incubator under 37°C and 5% CO_2_ conditions. The ADR-resistant cells (U87/ADR and U251/ADR) were obtained by adding an increasing concentration of ADR into the medium. The ADR concentration from 10 nM to 2 *μ*M. The ADR-resistant cell line was successfully constructed when the cells tolerated 2 *μ*M concentration of ADR, and the proliferation ability was similar to the wild-type cells. The culture medium of U87/ADR and U251/ADR cells was added to ADR (2 *μ*M/L) to maintain the ADR-resistant property.

### 2.3. Cell Transfection

Lentivirus vectors expressing MAPRE1, negative empty vector, and NC mimics/miR-526b-3p mimics were constructed from GeneChem (Shanghai, China). According to the instruction, each of the previously mentioned plasmids was transfected into cells for 48 h via Lipofectamine 6000 (Thermo Fisher Scientific, MA, USA).

### 2.4. Real-Time Quantitative Polymerase Chain Reaction (RT-qPCR)

Clinical tissues and cells were used to extract total RNA by utilizing Trizol reagent (Invitrogen, MA, USA), which was further reverse-transcribed to cDNA using PrimeScript RT reagent Kit (Takara, Japan). The reaction procedure of RT-qPCR complied with the instruction. In addition, U6 or *β*-actin was normalized by the 2^−ΔΔCT^ approach. The RT-qPCR primers are displayed in Table 1.

### 2.5. Cell Viability

CCK8 kit was used to analyze the cell viability. Briefly, transfected U87 and U251 cells were cultured in a 10% FBS medium until the logarithmic growth phase. Next, transfected U87/ADR and U251/ADR cells were cultured in a medium that was separately containing 0, 1, 2, 4, 8, and 16 *μ*M ADR for 48 h. Then, cells with a density of 10^3^ cells/100 *μ*L were inoculated in 96-well plates. CCK-8 kit (Sigma, USA) was utilized. Absorbance (OD) (450 nm) was detected using a spectrophotometer (Molecular Devices, San Jose, USA).

### 2.6. Colony Formation Assay

The colony formation assay was performed to assess the proliferative potential of transfected U87 and U251 cells. Briefly, cells were cultured within 6-well plates (500 cells), and the medium (Procell Life Science & Technology Co., Ltd.) was replaced every two to three days for a total of two weeks. Subsequently, cells were stained with 0.5% crystal violet for 15 min for 1 h at room temperature. Colonies (>50 cells) were observed and counted under a light microscope (Nikon, Japan, ×40).

### 2.7. Flow Cytometry

Cells from different groups were collected and washed twice with prechilled PBS and resuspended in a binding buffer. Then, the cells were stained with 5 *μ*L of Annexin V-FITC, and 10 *μ*L of PI was utilized to treat cells for 15 min in the dark according to the protocol. A flow cytometer (BD Biosciences, USA) was utilized for determining apoptotic cells.

### 2.8. Transwell Assay

Transwell migration and invasion assays (Corning, Inc.) were performed to assess transfected U87 and U251 cells' migratory and invasive abilities. Cells were cultured into the upper compartment with the basal medium in the migration assay. Lower chambers were supplied with 600 *μ*L medium containing 10% FBS. After 48 h, and cells were immobilized with methanol and stained with 0.1% crystal violet and observed under a microscope (Leica, Germany) and counted. In invasion assay, the used membrane was precoated with the Matrigel (Franklin Lakes, NJ, USA). The other methods were like cell migration. The migratory and invasive cells were counted under an IX70 inverted optical microscope (magnification, x100; Olympus Corporation).

### 2.9. Western Blot

Protein was extracted from glioma cells and measured through the BCA kit (Beyotime Biotechnology, China) [18]. Then, the protein was extracted using SDS-PAGE (10%) and transformed into PVDF membranes (Millipore, USA). Afterwards, membranes were incubated using 5% skimmed milk and incubated with primary antibodies under 4°C overnight. The antibodies are as follows: anti-Bax (1: 2, 000, bs-28034R, Bioss, China), anti-Bcl-2 (1: 2, 000, bs-4563R, Bioss, China), anti-MMP-2 (1: 2, 000, bs-20705R, Bioss, China), anti-MMP-9 (1: 2, 000, bs-22502R, Bioss, China), anti-Cleaved caspase-3 (1: 2, 000, bsm-33199M, Bioss, China), anti-Cleaved caspase-9 (1: 2, 000, bs-3082R, Bioss, China), anti-Cox-2 (1: 2, 000, bs-10411R, Bioss, China), and anti-*β*-actin (1: 2, 000, bs-0061R, Bioss, China) with *β*-actin being the endogenous control. Afterwards, membranes were incubated for 1 h using a secondary antibody (1: 2, 000, bs-0311P-HRP, Bioss). Finally, ECL (Millipore, USA) was utilized to observe protein blots and quantified using ImageJ software (version 4.3; National Institutes of Health).

### 2.10. Luciferase Reporter Assay

Wild-type luciferase reporter plasmid pGL3-MAPRE1-3′UTR (MAPRE1 WT) was constructed by General Bio (Anhui, China). Meanwhile, the MAPRE1 mutant luciferase reporter plasmid (MAPRE1 Mut) containing the mutation sequence of miR-526b-3p target binding site was constructed to confirm the binding specificity. First, U87 and U251 cells about 10^5^ were seeded into a 6-well plate and cultured 24 h. Then, the luciferase reporter plasmid (MAPRE1WT or MAPRE1 Mut), *β*-galactosidase plasmid, and mimics (NC mimics or miR-526b-3p mimics) were cotransfected. After 48 h-incubation at 37°C, the relative luciferase activity was measured (Promega, USA).

### 2.11. RNA Pull-Down Assay

Probes, including biotinylated miR-526b-3p WT and biotinylated miR-526b-3p Mut, or control probes purchased from RiboBio (Guangzhou, China) were transfected into U87 and U251 cells. Then, DNaseI was added to RNA solution, and incubation was for 5 min at 65°C. Afterwards, streptavidin-coated magnetic beads (New England BioLabs, USA) were incubated for another 4 h. Finally, the beads were washed with PBS and extracted RNA. MiR-526b-3p enrichment was detected via RT-qPCR analysis [19].

### 2.12. Statistical Analysis

The average ± standard deviation (SD) represented data from three repetitions, further analyzing using GraphPad Prism 5.0. Differences between groups were compared by ANOVA and Tukey's post hoc analysis. *P* < 0.05 was considered statistically significant.

## 3. Results

### 3.1. MiR-526b-3p Levels Are Decreased in Glioma and ADR-Resistant Glioma

**Figure 1:**
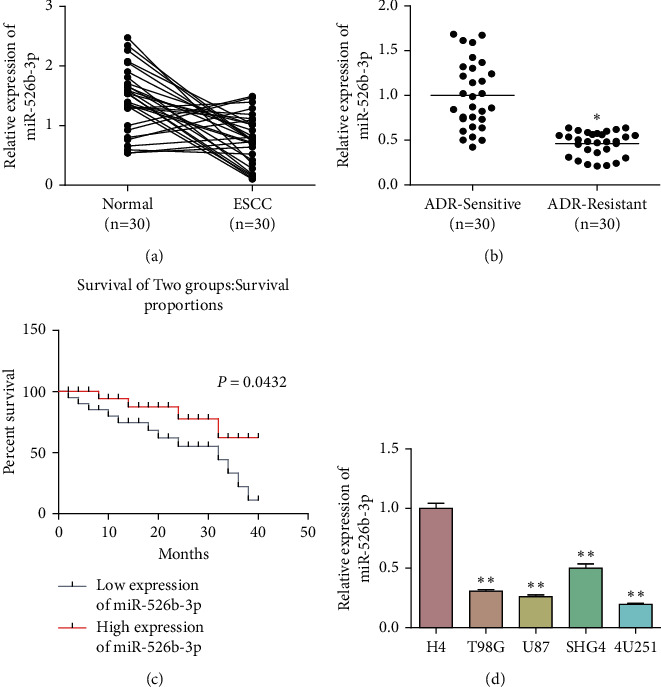
MiR-526b-3p expression in glioma and ADR-resistant glioma. RT-qPCR was used to detect the expression of miR-526b-3p in glioma and adjacent normal tissues (a) or ADR nonresistant and ADR-resistant glioma tissues (b). (c) The overall survival rates of glioma patients with miR-526b-3p high expression and low expression group. (d) The expression of miR-526b-3p in control cells H4 and glioma cells T98G, U87, SHG44, and U251. ^*∗∗∗*^ < 0.001, ^*∗∗*^*P* < 0.01, ^*∗*^*P* < 0.05. ^∗^indicates the difference with the control group. Error bars were represented the mean ± SD of triplicate experiments.

To explore miR-526b-3p functions on glioma to ADR resistance, miR-526b-3p levels within glioma and ADR-resistant glioma tissues were assessed through RT-qPCR. According to Figure 1(a), miR-526b-3p levels were reduced in glioma tissues compared with adjacent normal tissues. Compared with the ADR nonresistant glioma tissues, miR-526b-3p levels were decreased in ADR-resistant glioma tissues (Figure 1(b)). The survival analysis displayed that miR-526b-3p expression was positively correlated with the overall survival (Figure 1(c)). In addition, miR-526b-3p levels within glioma cells were determined. Figure 1(d) suggested that miR-526b-3p levels were downregulated within glioma cells (T98G, U87, SHG44, and U251) compared with the nonneoplastic H4 glioma cells (Figure 1(d)). These results suggested that miR-526b-3p participated in glioma development and ADR resistance.

### 3.2. MiR-526b-3p Affects the Pathological Process of Glioma

**Figure 2:**
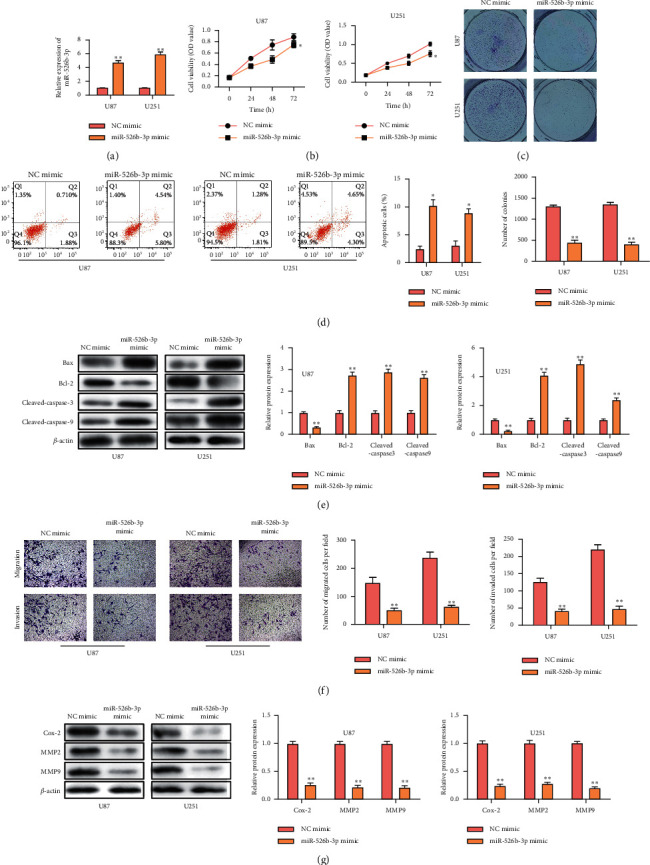
The effect of miR-526b-3p overexpression in the glioma process. U87 and U251 cells transfected miR-526b-3p mimics or NC mimics separately. (a) miR-526b-3p expression was detected by RT-qPCR. (b) CCK8 assay detected the cell viability. (c) Colony formation was detected in transfected cells. (d) Flow cytometry detected cell apoptosis difference in miR-526b-3p upregulated glioma cells. (e) Western blot was used to measure cell apoptosis-related genes expression. (f) The cell migration and cell invasion were detected through a Transwell assay. (g) Western blot was used to detect the genes expression of cell migration and invasion in protein levels. ^*∗∗∗*^ < 0.001, ^*∗∗*^*P* < 0.01, ^*∗*^*P* < 0.05. ^∗^indicates the difference with the NC mimics-transfected cells. Error bars were represented the mean ± SD of triplicate experiments.

To determine miR-526b-3p roles in the glioma process, miR-526b-3p mimics and NC mimics were transfected into U87 and U251 cells. MiR-526b-3p upregulation was confirmed using RT-qPCR (Figure 2(a)). Based on CCK-8, cell proliferation was attenuated in miR-526b-3p mimics-transfected cells, consistent with the colony formation assay results (Figures 2(b) and 2(c)). According to flow cytometry analysis, Figure 2(d) showed cell apoptosis rate was increased within miR-526b-3p mimics-transfected cells (Figure 2(d)). The apoptosis-associated genes expression was detected, and the results indicated that miR-526b-3p promoted cell apoptosis of glioma (Figure 2(e)). The Transwell assay results showed that the migration and invasion of miR-526b-3p overexpressed cells were lower than the control cells (Figure 2(f)). Cell migration and invasion are associated with gene expression at protein levels, which was detected by western blot. As displayed in Figure 2(g), miR-526b-3p overexpression inhibited the migration and invasion gene expressions (Figure 2(g)). These results demonstrated that miR-526b-3p overexpression inhibited glioma cell proliferation, migration, and invasion while promoting cell apoptosis.

### 3.3. MiR-526b-3p Inhibits the Resistance of Glioma to ADR

**Figure 3:**
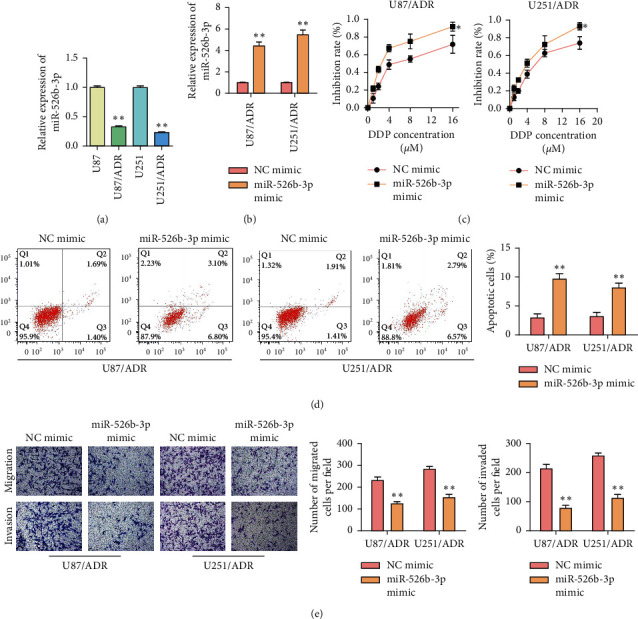
Overexpression of miR-526b-3p inhibits the resistance of glioma to ADR. (a) RT-qPCR detected the expression of the miR-526b-3p in U87/ADR and U251/ADR cells compared with normal glioma cells. (b) miR-526b-3p expression in U87/ADR and U251/ADR cells that transfected miR-526b-3p mimics or NC mimics separately. (c) CCK8 assay detected the miR-526b-3p effect of ADR resistance in U87/ADR and U251/ADR cells. (d) Flow cytometry detected cell apoptosis difference in miR-526b-3p upregulated U87/ADR and U251/ADR cells. (e) Transwell assay detected cell migration and invasion difference in miR-526b-3p overexpression ADR-resistant glioma cells. ^*∗∗∗*^ < 0.001, ^*∗∗*^*P* < 0.01, ^*∗*^*P* < 0.05 vs. the nonresistant cells or transfected NC mimics ADR-resistant cells. Error bars were represented the mean ± SD of triplicate experiments.

ADR-resistant cells (U87/ADR and U251/ADR) were applied to investigate miR-526b-3p functions on ADR resistance of glioma. MiR-526b-3p levels within U87/ADR and U251/ADR cells were detected firstly.  Figure 3(a) showed that miR-526b-3p levels were reduced in ADR-resistant cells compared with common glioma cells. Then, miR-526b-3p mimics were transfected into U87/ADR and U251/ADR cells. The transfection efficiency was detected, and the results are shown in Figure 3(b). Transfected cells were cultured for 48 h with 0, 1, 2, 4, 8, and 16 *μ*M ADR, and the cell proliferation inhibition ratio was detected.  Figure 3(c) illustrated that miR-526b-3p overexpression reduced the IC_50_ of U87/ADR and U251/ADR cells significantly. Besides, miR-526b-3p overexpression promoted U87/ADR and U251/ADR cells apoptosis (Figure 3(d)). Transwell assay confirmed that the miR-526b-3p overexpression suppressed U87/ADR and U251/ADR cell migration and invasion (Figure 3(e)). These results suggested that miR-526b-3p overexpression inhibited glioma cell resistance to ADR.

### 3.4. MAPRE1 Is a Downstream Target Gene of miR-526b-3p

**Figure 4:**
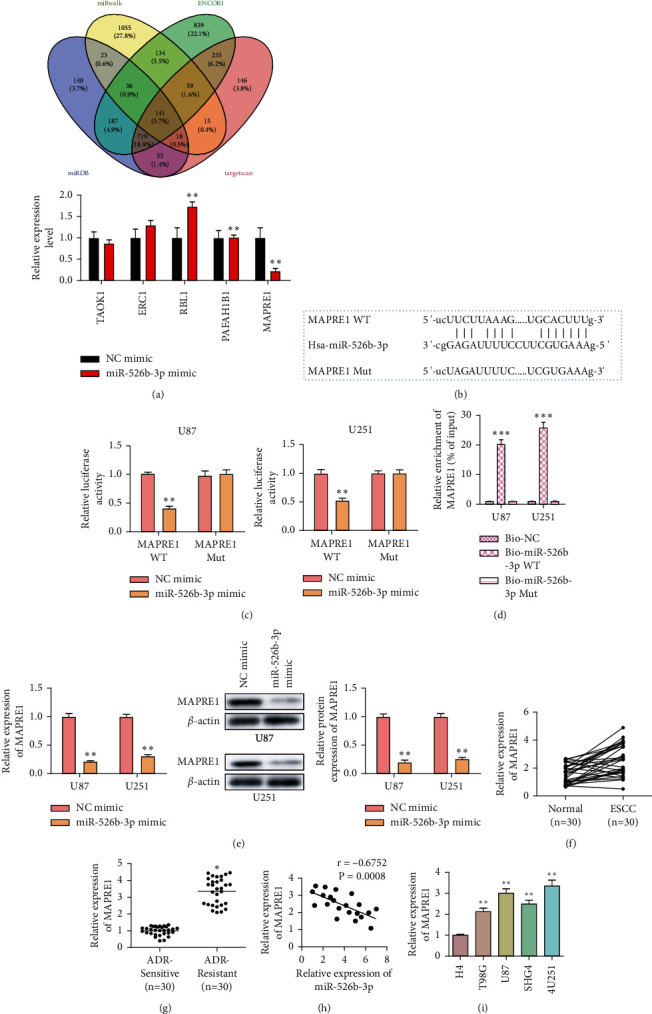
MAPRE1 is a downstream target gene of miR-526b-3p. (a) Prediction of the target genes of miR-526b-3p and RT-qPCR detected these genes expression in normal and glioma cells. (b) The putative target sequence of miR-526b-3p in MAPRE1 3′UTR and mutated sequence. (c) Luciferase signal was detected in U87 and U251 cells transfected with wild-type or mutant MAPRE1, and miR-526b-3p mimics or NC mimics. (d) Pull-down assay detected the miR-526b-3p target binding the MAPRE1 in U87 and U251 cells. (e) RT-qPCR and western blot detected the MAPRE1 expression in U87 and U251 cells that transfected miR-526b-3p mimics or NC mimics. (f) RT-qPCR detected MAPRE1 expression in glioma and adjacent normal tissues. (g) RT-qPCR detected MAPRE1 expression in ADR nonresistant and resistant glioma tissues. (h) The expression correlation between miR-526b-3p and MAPRE1 in glioma tissues. (i) RT-qPCR detected MAPRE1 expression in glioma cells. ^∗∗∗^<0.001, ^∗∗^*P* < 0.01, ^∗^*P* < 0.05. ^∗^indicates the difference with the control group. Error bars were represented the mean ± SD of triplicate experiments.

MiRNAs mainly exert their regulatory abilities by regulating the expression of targeted RNA. To elucidate the molecular mechanisms of miR-526b-3p in glioma, the target genes of miR-526b-3p were predicted using the ENCORI, miRwalk, and TargetScan, and RT-qPCR detected these gene expressions. As shown in Figure 4(a), only MAPRE1 was dramatically elevated in glioma cells compared with other genes. The potential binding site of MAPRE1 and miR-526b-3p was displayed in Figure 4(b). To validate the target binding, a luciferase assay was performed. The data of Figure 4(c) showed that miR-526b-3p upregulation reduced the luciferase activity in MAPRE1 WT-transfected cells. However, the luciferase activity in MAPRE1 Mut-transfected cells had no significant change (Figure 4(c)). Pull-down assay results also displayed that MAPRE1 bound with miR-526b-3p WT rather than miR-526b-3p Mut (Figure 4(d)). Then, we detected MAPRE1 expression at the RNA and protein levels in miR-526b-3p overexpression cells. The results showed that MAPRE1 levels were decreased within miR-526b-3p overexpressed cells compared with control cells (Figure 4(e)). Further, MAPRE1 levels within glioma tissues and ADR-resistant glioma tissues were analyzed. Based on Figures 4(f) and 4(g), MAPRE1 levels were increased in glioma tissues and ADR-resistant glioma tissues. The expression correlation analysis showed that MAPRE1 expression was negatively regulated by miR-526b-3p (Figure 4(h)). Consistently, increased expression of MAPRE1 in glioma cells was confirmed (Figure 4(i)). These findings demonstrated that miR-526b-3p targeted binding to MAPRE1 and negatively regulated MAPRE1 expression in glioma.

### 3.5. MiR-526b-3p Inhibits the Process and ADR Resistance of Glioma through MAPRE1

**Figure 5:**
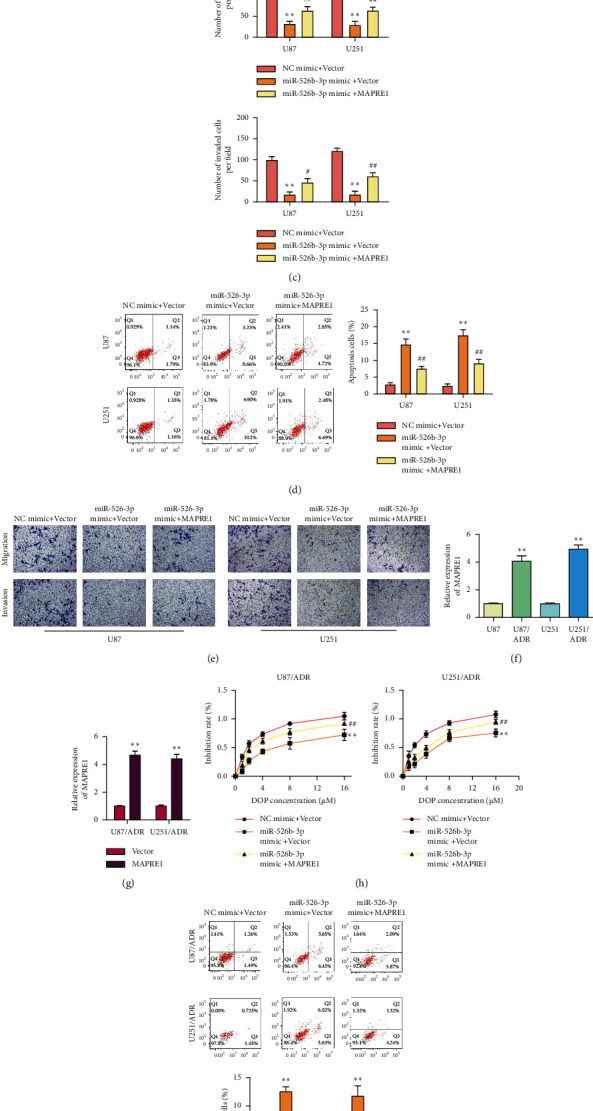
MiR-526b-3p inhibits the development and ADR resistance of glioma through MAPRE1. U87 and U251 cells transfected MAPRE1 vector or control vector, respectively. The efficiency of MAPRE1 overexpression was detected by RT-qPCR. (a) U87 and U251 cell transfected miR-526b-3p mimics and NC vector or transfected miR-526b-3p mimics and MAPRE1 vector simultaneously. Transfect NC mimics and control vector group cells were used as the negative control. (b) Cell viability was detected by CCK8 assay. (c) Colony formation assay detected cell proliferation. (d) Flow cytometry detected cell apoptosis ratio. (e) Cell migration and cell invasion were detected through a Transwell assay. (f) RT-qPCR detected the expression of MAPRE1 in ADR nonresistant and ADR-resistant U87 and U251 cells. (g) The expression of MAPRE1 in U87/ADR and U251/ADR cells that transfected the MAPRE1 vector or control vector. U87/ADR and U251/ADR cells transfected miR-526b-3p mimics and NC vector or miR-526b-3p mimics and MAPRE1 vector simultaneously. Cell inhibition ratio (h), cell apoptosis ratio (i), cell migration, and cell invasion (j) were detected in transfected ADR-resistant cells. ^∗∗∗^<0.001, ^∗∗^*P* < 0.01, ^∗^*P* < 0.05*vs* the NC mimics and control vector cotransfected cells. ^###^*P* < 0.001, ^##^*P* < 0.01 P, ^#^*P* < 0.05 vs. the miR-526b-3p mimics and control vector cotransfected cells. Error bars were represented the mean ± SD of triplicate experiments.

To further verify the miR-526b-3p regulate glioma process through MAPRE1, we transfected the MAPRE1 vector in U87 and U251 cells. RT-qPCR detected the transfection efficiency, and the results are shown in Figure 5(a). MiR-526b-3p mimics and MAPRE1 vector were cotransfected in U87 and U251 cells. Cell viability assay and clone formation assay results suggested that the overexpression of MAPRE1 retarded the inhibition of cell proliferation induced by miR-526b-3p mimics (Figures 5(b) and 5(c)). Similarly, the increased cell apoptosis ratio induced by miR-526b-3p has attenuated in MAPRE1 overexpressed cells (Figure 5(d)). Transwell assay results also suggested that MAPRE1 rescued the inhibition effects of miR-526b-3p on cell migration and invasion (Figure 5(e)). These results clarified that miR-526b-3p regulated the glioma process through MAPRE1.

To verify the miR-526b-3p regulating glioma resistance to ADR through MAPRE1, MAPRE1 expression in ADR-resistant cells was detected first. The results showed that MAPRE1 expression was increased in ADR-resistant glioma cells compared with the normal glioma cells (Figure 5(f)). Then, we transfected the MAPRE1 vector separately or transfected miR-526b-3p mimics and MAPRE1 vector simultaneously in U87/ADR and U251/ADR cells. The transfection efficiency of the MAPRE1 vector is shown in Figure 5(g). The cotransfected cells were cultured with different concentrations of ADR. After 48 h, the cell proliferation inhibition ratio was assessed by CCK8. The results showed that MAPRE1 overexpression restored the cell inhibition ratio induced by miR-526b-3p upregulation (Figure 5(h)). In line with the CCK8 assay results, flow cytometry results showed that MAPRE1 retarded the increase of apoptosis rate induced by miR-526b-3p within ADR-resistant glioma cells (Figure 5(i)). Transwell assay results showed that MAPRE1 rescued the migration and invasion roles of miR-526b-3p in ADR-resistant glioma cells (Figure 5(j)). These findings demonstrated that miR-526b-3p inhibited the glioma resistance to ADR by downregulating the expression of MAPRE1.

## 4. Discussion

Resistance is the primary clinical obstacle to successfully treating cancer, including glioma [20]. The causes of tumor resistance are very complex. The abnormal expression of miRNAs has been reported to correlate with tumor resistance, such as exosomes secreted by tumor-associated M2 macrophages transferred miR-21 to the gastric cells. High levels of miR-21 promote the cisplatin resistance of gastric cancer [21]. In lung cancer, miR-146a has been proven not to affect the growth of the tumor but to alter chemotherapy sensitivity by regulating the CHOP expression [22]. Many reports confirmed that miRNAs expression dysregulation was involved in the process of glioma resistance. For instance, miR-130a-3p expression decreased in glioblastoma cell lines, and the target regulates the expression of Sp1. Increased levels of miR-130-3p inhibit the proliferation and TMZ resistance of glioblastoma cells [23]. Li et al. found that miR-186 inhibits the glioblastoma resistance to cisplatin by downregulating the expression of YY1. MiR-186 inhibits glioblastoma-initiating cell (GIC) phenotype formation in U87MG cells [24]. MiR-526b-3p has been reported to regulate multiple tumor progression [17, 25]. For instance, upregulation of miR-526b-3p inhibits cell viability, migration, and tube formation of glioma-associated endothelial cells [26].

Nevertheless, miR-526b-3p roles in tumor resistance have not been reported. So, the functions of miR-526b-3p in glioma progress and ADR resistance were investigated. As expected, miR-526b-3p levels were decreased in glioma tissues and cells. Meanwhile, in ADR-resistant glioma tissues and cells, miR-526b-3p levels were also reduced. Upregulating miR-526b-3p levels repressed the proliferation, metastasis, and ADR resistance while promoting apoptosis in glioma cells. Further, we explored the regulatory signal pathway of miR-526b-3p in glioma. We found that miR-526b-3p targeted binding to MAPRE1 and negatively regulated the expression of MAPRE1.

As an RP/EB family member, MAPRE1 encoded microtubule-associated protein. It has been reported that MAPRE1 is involved in mitochondrial membrane degradation [27]. Moreover, MAPRE1 works as a diagnosis and prognosis biomarker in cancer [28]. High levels of MAPRE1 are correlated with tumor malignancy, high histological grade, and poor clinical outcome [29]. Besides, Kim et al. found that MAPRE1 promotes cell apoptosis via reactive oxygen species, and Bax mediates mitochondrial dysfunction in NSCLC [30]. Wang et al. found that MAPKE1 overexpresses in ESCC and promotes cellular growth by affecting suppressor gene APC function and activating the beta-catenin/TGF pathway [31]. However, the relationship between MAPKE1 and tumor resistance remained unknown. In this study, MAPKE1 expression was increased in glioma tissues and cells. We also proved that MAPKE1 levels were increased in ADR-resistant glioma tissues and cells. Overexpression of MAPKE1 restored the miR-526b-3p-induced change in the glioma process and ADR resistance.

However, the present study had some limitations. For example, fluorescence in situ hybridization (FISH) could better detect the distribution and binding relationship between miR-526b-3p and MAPRE1. Furthermore, further in vivo experiments confirmed that miR-526b-3p increased the sensitivity of glioma cells to ADRs in vivo, which could increase the scientific relevance of this study.

In summary, this study proved that miR-526b-3p exerted a vital role in glioma development and ADR resistance. Furthermore, overexpression miR-526b-3p inhibited tumor procession and increased the sensitivity of glioma to ADR through downregulating the expression of MAPRE1. These results suggested that miR-526b-3p functioned as the diagnostic marker in glioma development and the potential treatment target of the glioma resistance to ADR. At the same time, these results can provide a new treatment strategy and experimental basis for glioma resistance to ADR.

## Figures and Tables

**Table 1:** Primers were used for RT-qPCR.

Gene name	Forward primer (5′-3′)	Reverse primer (5′-3′)
miR-526b-3p	GAAAGUGCUUCCUUUUAGAGGC	GCCTCTAAAAGGAAGCACTTTC
U6	GCTTCGGCAGCACATATACT	AACGCTTCACGAATTTGCGT
MAPRE1	CTGGCGGTTTGAACGAGAC	CACTGCCATCTTCGGCTCT
GAPDH	TCTCTGCTCCTCCTGTTC	GTTGACTCCGACCTTCAC

## Data Availability

All data generated or analyzed during this study are included in this published article.

## References

[1] Ostrom Q. T., Gittleman H., Stetson L., Virk S., Barnholtz-Sloan J. S. (2018). Epidemiology of intracranial gliomas. *Progress in Neurological Surgery*.

[2] Lin L., Cai J., Jiang C. (2017). Recent advances in targeted therapy for glioma. *Current Medicinal Chemistry*.

[3] Brown T. J., Bota D. A., van Den Bent M. J. (2019). Management of low-grade glioma: a systematic review and meta-analysis. *Neuro-Oncology Practice*.

[4] Tomiyama A., Ichimura K. (2019). Signal transduction pathways and resistance to targeted therapies in glioma. *Seminars in Cancer Biology*.

[5] Yuan C., Kulkarni K., Dashevsky B. Z. (2020). Preventive care: how mammography utilization changes as women age. *Journal of the American College of Radiology*.

[6] Saliminejad K., Khorram Khorshid H. R., Soleymani Fard S., Ghaffari S. H. (2019). An overview of microRNAs: biology, functions, therapeutics, and analysis methods. *Journal of Cellular Physiology*.

[7] Javanmardi S., Aghamaali M., Abolmaali S., Mohammadi S., Tamaddon A. (2017). miR-21, an oncogenic target miRNA for cancer therapy: molecular mechanisms and recent advancements in chemo and radio-resistance. *Current Gene Therapy*.

[8] Bayraktar R., Van Roosbroeck K. (2018). miR-155 in cancer drug resistance and as target for miRNA-based therapeutics. *Cancer and Metastasis Reviews*.

[9] Park C. H., AyeThwe A., Kim S. J. (2016). Effect of auxins on anthocyanin accumulation in hairy root cultures of tartary buckwheat cultivar hokkai T1O. *Natural Product Communications*.

[10] Zhang X., Yu J., Zhao C. (2019). MiR-181b-5p modulates chemosensitivity of glioma cells to temozolomide by targeting Bcl-2. *Biomedicine & Pharmacotherapy*.

[11] Wang H., Ren S., Xu Y. (2019). MicroRNA-195 reverses the resistance to temozolomide through targeting cyclin E1 in glioma cells. *Anti-Cancer Drugs*.

[12] Ma W., Cui Y., Liu M., Tan Z., Jiang Y. (2019). Downregulation of miR-125b promotes resistance of glioma cells to TRAIL through overexpression of tafazzin which is a mitochondrial protein. *Aging*.

[13] Siles L., Alegre L., Tijero V., Munné-Bosch S. (2015). Enhanced tocopherol levels during early germination events in *Chamaerops humilis* var. humilis seeds. *Phytochemistry*.

[14] Jiang L., Shi S., Shi Q., Zhang H., Xia Y., Zhong T. (2018). MicroRNA-519d-3p inhibits proliferation and promotes apoptosis by targeting HIF-2*α* in cervical cancer under hypoxic conditions. *Oncology Research Featuring Preclinical and Clinical Cancer Therapeutics*.

[15] Diep G. K., Adams J. E. (2016). The prodrome of extensor pollicis longus tendonitis and rupture: rupture may be preventable. *Orthopedics*.

[16] Fang Z., Yang H., Chen D. (2019). YY1 promotes colorectal cancer proliferation through the miR-526b-3p/E2F1 axis. *American Journal of Cancer Research*.

[17] Wu M., Li X., Liu Q., Xie Y., Yuan J., Wanggou S. (2019). miR-526b-3p serves as a prognostic factor and regulates the proliferation, invasion, and migration of glioma through targeting WEE1. *Cancer Management and Research*.

[18] Peng X. P., Huang L., Liu Z. H. (2016). miRNA-133a attenuates lipid accumulation via TR4-CD36 pathway in macrophages. *Biochimie*.

[19] Liu Y., Liu R., Yang F. (2017). miR-19a promotes colorectal cancer proliferation and migration by targeting TIA1. *Molecular Cancer*.

[20] Noch E. K., Ramakrishna R., Magge R. (2018). Challenges in the treatment of glioblastoma: multisystem mechanisms of therapeutic resistance. *World Neurosurgery*.

[21] Zheng P., Chen L., Yuan X. (2017). Exosomal transfer of tumor-associated macrophage-derived miR-21 confers cisplatin resistance in gastric cancer cells. *Journal of Experimental & Clinical Cancer Research*.

[22] Tan W., Liao Y., Qiu Y. (2018). miRNA 146a promotes chemotherapy resistance in lung cancer cells by targeting DNA damage inducible transcript 3 (CHOP). *Cancer Letters*.

[23] Wang Z., Li Z., Fu Y., Han L., Tian Y. (2019). MiRNA-130a-3p inhibits cell proliferation, migration, and TMZ resistance in glioblastoma by targeting Sp1. *American Journal of Translational Research*.

[24] Li J., Song J., Guo F. (2019). miR-186 reverses cisplatin resistance and inhibits the formation of the glioblastoma-initiating cell phenotype by degrading Yin Yang 1 in glioblastoma. *International Journal of Molecular Medicine*.

[25] Yan F., Ma Y., Liu L., Li L., Deng J., Sun J. (2020). Long noncoding RNA HOXD-AS1 promotes the proliferation, migration, and invasion of colorectal cancer via the miR-526b-3p/CCND1 axis. *Journal of Surgical Research*.

[26] Liu X., Shen S., Zhu L. (2020). SRSF10 inhibits biogenesis of circ-ATXN1 to regulate glioma angiogenesis via miR-526b-3p/MMP2 pathway. *Journal of Experimental & Clinical Cancer Research*.

[27] Wang Y. H., Wang J. Q., Wang Q. (2016). Endophilin B2 promotes inner mitochondrial membrane degradation by forming heterodimers with endophilin B1 during mitophagy. *Scientific Reports*.

[28] Taguchi A., Rho J. H., Yan Q. (2015). MAPRE1 as a plasma biomarker for early-stage colorectal cancer and adenomas. *Cancer Prevention Research*.

[29] Rodrigues-Ferreira S., Nehlig A., Monchecourt C. (2019). Combinatorial expression of microtubule-associated EB1 and ATIP3 biomarkers improves breast cancer prognosis. *Breast Cancer Research and Treatment*.

[30] Kim M. J., Yun H. S., Hong E. H. (2013). Depletion of end-binding protein 1 (EB1) promotes apoptosis of human non-small-cell lung cancer cells via reactive oxygen species and Bax-mediated mitochondrial dysfunction. *Cancer Letters*.

[31] Wang Y., Zhou X., Zhu H. (2005). Overexpression of EB1 in human esophageal squamous cell carcinoma (ESCC) may promote cellular growth by activating *β*-catenin/TCF pathway. *Oncogene*.

